# Oral Verruciform Xanthoma: A Case Report and Literature Review

**DOI:** 10.1155/2014/641015

**Published:** 2014-12-08

**Authors:** Yonara Maria Freire Soares Marques, Cleverton Roberto de Andrade, Suzana Cantanhede Orsini Machado de Sousa, Cláudia Maria Navarro

**Affiliations:** ^1^Department of Bioscience and Oral Diagnosis, Dental School, Institute of Science and Technology (ICT), Paulista State University (UNESP), 777 Engenheiro Francisco José Longo Avenue, 12245-000 São José dos Campos, SP, Brazil; ^2^Department of Physiology and Pathology, Paulista State University (UNESP), 1680 Humaitá Street, 14801-903 Araraquara, SP, Brazil; ^3^Department of Oral Pathology, Universitty of São Paulo (USP), 2227 Professor Lineu Prestes Avenue, 05508-000 São Paulo, SP, Brazil; ^4^Department of Diagnosis and Surgery, Paulista State University (UNESP), 1680 Humaitá Street, 14801-903 Araraquara, SP, Brazil

## Abstract

Oral verruciform xanthoma represents an uncommon entity, which affects mainly oral mucosa. This paper presents the major clinical and histological features of oral verruciform xanthoma and reports a case on the tongue. The differential diagnosis and a literature review are also provided in light of recent information.

## 1. Introduction

Oral verruciform xanthoma (OVX) is an uncommon benign lesion, which affects predominantly oral mucosa, usually presents a normal or reddish colour and sometimes pale or “hyperkeratotic” pattern, and has a rough, pebbly surface, with either sessile or pedunculated base, and diameter between 2 mm and 1.5 cm. The frequency of this lesion ranges from 0.025% to 0.094%, with a slight male predilection. Many cases have been reported in Asiatic patients and the mean age of occurrence is between 38.5 and 54.9 years [1, 2]. The etiology and pathological mechanism of the OVX development remain elusive so far.

The aim of this paper was to report an uncommon case of oral verruciform xanthoma and discuss the most recent findings about this lesion.

## 2. Case Report

**Figure 1:**
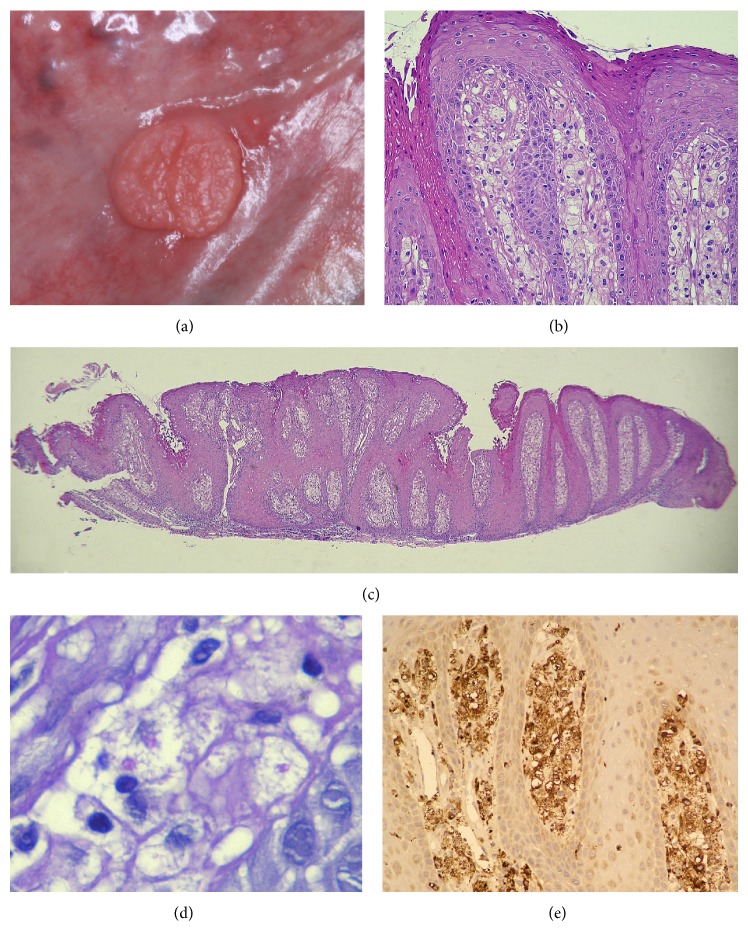
Clinical consistency of OVX with granular, yellow-whitish surface in the lateral border of the tongue (a); photomicrograph of OVX (H and E; 100x) showing the connective tissue exhibiting the accumulation of foam cell between the epithelial rete pegs (b); low magnification 10x of the lesion exhibiting the uniform rete pegs with parakeratotic invaginating crypts and connective tissue filled with xanthoma cells (c); negative image of fat and PAS-positive granules inside the cytoplasm (high magnification 400x) (d); and strong positive immunoreactivity to antibody CD-68 (high magnification 200x) (e).

A 73-year-old woman presented a 4-month-history of an asymptomatic soft tissue mass of the lateral edge of the tongue. Her past medical history was unremarkable. Physical examination of oral mucosa revealed a well-circumscribed, sessile nodule with slight pedunculation at the periphery and fibrous consistency and yellow-whitish verrucous surface fixed to the lateral edge of the tongue (Figure 1(a)). The nodule was about 0.5 cm in diameter. These findings were suggestive of condyloma acuminatum, verruca vulgaris, or giant cell fibroma. Excisional biopsy of the soft mass was performed and histopathological examination revealed a parakeratotic epithelium with mild acanthosis, uniform elongated epithelial ridges, with parakeratotic plugs, and exocytosis in superficial layer (Figure 1(c)). The connective tissue was composed by uniform papillae filled with large vacuolated foam cells (xanthoma cells) with eccentrically placed nuclei (Figure 1(b)). Furthermore, chronic inflammatory infiltration was found in the connective tissue underneath the epithelial projections. The Periodic Acid-Shiff (PAS) reaction exhibited positivity on granules inside the foam cells and immunohistochemical reaction to CD-68 antibody revealed a strong and uniform staining of all the subepithelial foamy macrophages (Figures 1(d) and 1(e)). These findings were consistent with the diagnosis of verruciform xanthoma.

## 3. Discussion

OVX is an uncommon lesion characterized by accumulation of foam cells in subepithelial mucosa. It has a significant predilection for oral mucosa. The mastigatory mucosa represents the most common site (85.3%) reported in the literature. However, other sites as floor of the mouth and labial mucosa have also been reported [1–3].

The origin of xanthoma cells remains unclear in the literature. Nowadays, many hypotheses have been proposed to explain the etiologic factors and pathogenic mechanisms involved with inflammatory, viral, and immunological disorders [4–6]. From a general point of view, these hypotheses could be justified, respectively, by cases often observed on mastigatory mucosa, which comprises area subjected to trauma and possibly followed by inflammatory reaction; few cases were reported in genital regions, which are commonly associated with viral infection, and also cases that occur in conjunction with diseases such as pemphigus vulgaris, lichen planus [7], psoriasis [8], and dystrophic epidermolysis bullosa [9], corresponding to lesions related to immunological reaction. However, these associations remain without consistent explanation.

The most recent studies have analyzed the foam cells of OVX in an attempt to clarify the immunohistochemical/ultrastructural characterization and possible mechanism of migration of xanthoma cell to the subepithelial region.

Immunohistochemically, the foam cells from OVX have been characterized as originating from a macrophagic lineage due to the strong immunoreactivity to CD-68 antibody [3, 10]. In addition, using antibody probes to identify macrophages subpopulation, it was observed that verruciform xanthoma cells are predominantly composed by cells with reparative and mature-resistent phenotype (positive to RM3/1, 25F9 and 27E10), and limited presence of acute inflammatory cell type [6].

In relation to OVX pathogenic mechanism, study based on immunohistochemical and ultrastructural analysis suggested that, under synergistic regulation of T cells, there are a recruitment of MCP-1/CCR2-mediated macrophage in the subbasal papillae and the lysosomal engulfment of epithelial lipids by MSR-I-bearing macrophages, and this mechanism may play a central role in pathogenesis of OVX. The foam cell necrosis and macrophages-dependent debris disposal may keep the macrophage recruitment under control after OVX developed [10].

Clinically, OVX usually presents as an isolated, asymptomatic, and pink to gray nodule but occasionally exhibits a yellow surface. The surface can present a papillary/granular or verrucous aspect with a sessile or pedunculated base [2].

The typical histological findings of OVX are a papillary or verrucous proliferation of stratified squamous epithelium associated with acanthosis and hyperkeratosis. The superficial parakeratotic layer can be brightly eosinophilic with cell desquamating on it and can form some invaginating crypts extending into the epithelium, sometimes exhibiting keratotic plugs. The epithelium can extend as relatively uniform elongated rete pegs into the connective tissue. The connective tissue papillae between the rete pegs are characterized by massive accumulation of large swollen foam cells, which are restricted to the extension of the rete pegs. The cytoplasm of the foam cells contains abundant negative image of lipids and tiny PAS-positive granule. The nuclei are small or round and eccentrically placed [3].

Due to the nonspecific clinical aspect of OVX, the clinical differential diagnosis usually includes lesions with similar characteristics especially the rough surface, such as squamous papiloma, verruga vulgaris, condyloma acuminatum, verrucous leukoplakia, and verrucous carcinoma [2].

The treatment of OVX consists of surgical excision and recurrence is extremely rare [1].

## 4. Conclusions

In spite of the very few reports of OVX, the clinicians should be familiar with clinical and histological features of this lesion to avoid unnecessary extensive surgical procedures due to the similarity to other lesions as verrucous carcinoma. In addition, OVX should be considered in differential diagnoses of solitary verrucous lesion in oral mucosa.
